# Adenocarcinoma Metastasis from Colon to the Thyroid

**DOI:** 10.1155/2022/8705143

**Published:** 2022-11-07

**Authors:** Parviz Mardani, Nazanin Ayareh, Hooman Kamran, Reza Shahriarirad, Masoud Vafabin, Neda Soleimani

**Affiliations:** ^1^Thoracic and Vascular Surgery Research Center, Shiraz University of Medical Science, Shiraz, Iran; ^2^Students Research Committee, School of Medicine, Shiraz University of Medical Sciences, Shiraz, Iran; ^3^Department of Pathology, Shiraz Transplant Center, Abu Ali Sina Hospital, Shiraz University of Medical Sciences, Shiraz, Iran; ^4^Department of Pathology, Shiraz University of Medical Sciences, Shiraz, Iran

## Abstract

**Background:**

Colorectal cancer metastasis to the thyroid is extremely rare and happens in the late course of the disease. *Case Description.* Here is the report of a 55-year-old female patient who came to us with the chief complaint of cough, diagnosed with colon metastasize to the lung. Surgical resection of the mass was performed. However, a thyroid mass was found incidentally in her postoperative follow-up. Fine needle aspiration of thyroid mass showed papillary thyroid carcinoma. But, after thyroidectomy, the origin of the mass was reported to be adenocarcinoma metastasis from colon cancer.

**Conclusion:**

Although thyroid metastasis from colorectal cancer rarely occurs, it should be considered in a patient with a solitary thyroid nodule and a past medical history of colon cancer. Surgical treatment is the preferred choice of treatment in these cases.

## 1. Introduction

Colon cancer is among the most common cancers worldwide, with a high propensity to metastasize, mostly spreading to the liver, lung, peritoneum, and regional lymph nodes [1]. Thyroid metastasis occurs in around 1.5–7.5% of all thyroid malignancies [2], while metastasis originating from the colon has rarely been reported [3]. Since, thyroid metastasis does not respond to treatment very well, their prognosis is considered poor; however, novel targeted and molecular therapeutic approaches have been developed in recent years, which require a thorough investigation of the mechanisms of tumor resistance and analysis of acquired second hit mutations, to tailor therapy against the changing of the molecular landscape of the tumor over time [4].

In general, cancer metastasis to the thyroid is rare; furthermore, metastasis from colorectal cancer to the thyroid gland is extremely rare and happens in the late course of the disease. In a study by Liévre et al., which was performed on 5862 colorectal cancer patients from 1993 to 2004, only 6 patients were found to have thyroid metastasis [2]. Here, we present a 55-year-old female patient with an adenocarcinoma metastasis from previous colon cancer to the thyroid.

## 2. Case Description

A 55-year-old female with a previous history of colon cancer was referred to our center with the chief complaint of dry cough for the past two months. In physical examination, she had stable vital signs with an oxygen saturation of 95%.

**Figure 1:**
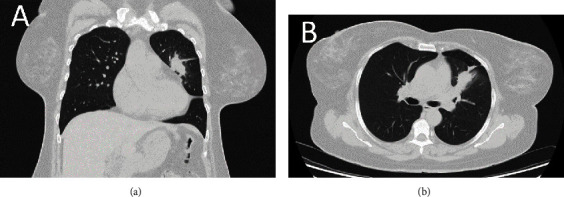
Computed tomography scan showing a mass in the upper part of the left lung: (a) coronal view and (b) sagittal view.

Eight years before admission, she had undergone a left hemicolectomy due to colon cancer. In addition, she had received ten sessions of chemotherapy and ten sessions of radiotherapy following the surgery. So, dry cough was suspected to be due to metastasis to the lung. Computed tomography (CT) scan was performed, showing a mass in the upper part of the left lung (Figure 1). Therefore, the patient was scheduled for surgical resection.

Under general anesthesia, first, fiberoptic bronchoscopy was performed, showing no signs of any intrabronchial lesions. Then, the left upper lobectomy and mediastinal lymphadenectomy were done. The operation ended with the insertion of two chest tubes. The pathological evaluation reported metastatic adenocarcinoma, and the resected margin was free of tumor. The patient was discharged in good condition five days after the operation and follow-up for the next week.

**Figure 2:**
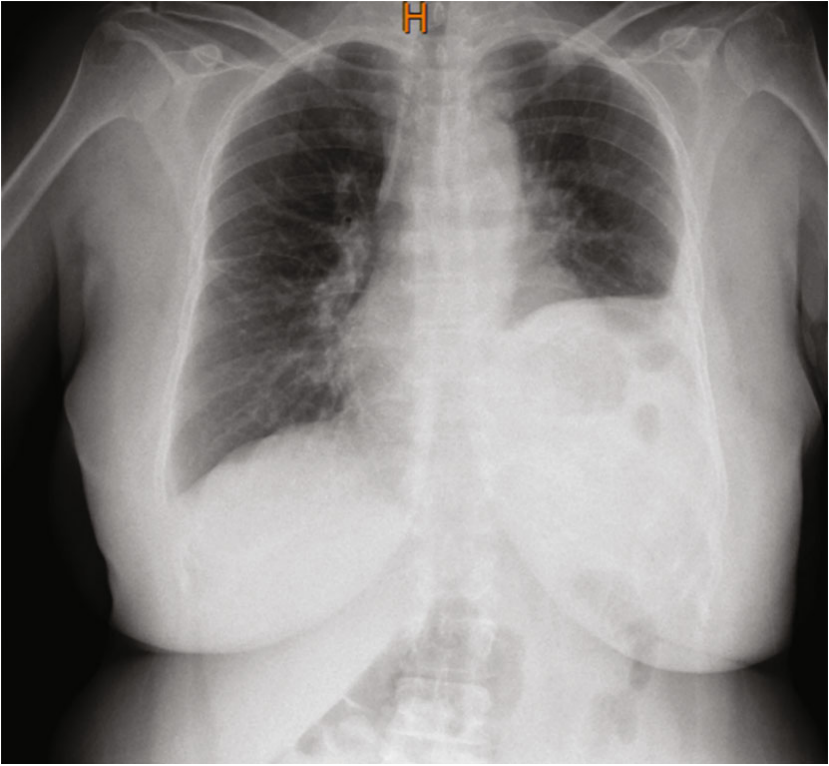
Chest X-ray of our patient on the day that she presented with dyspnea and chest pain.

In her follow-up, the patient came to our center with dyspnea and chest pain four days later (Figure 2). Oxygen saturation was 84% in room air. The initial diagnosis was pulmonary thromboembolism (PTE) vs. low serum calcium. Transthoracic echocardiography showed an ejection fraction of 60% with no evidence of PTE. Also, the total serum calcium level was 7.8 at admission. So, PTE was ruled out and the patient was discharged in good condition with calcium supplements and a follow-up of an endocrinologist.

The patient underwent a positron emission tomography (PET) scan, revealing the spreading of cancer to the thyroid. Fine needle aspiration (FNA) was performed, which showed papillary thyroid carcinoma. So, another operation was scheduled, and total thyroidectomy and central neck lymph node dissection were done. The mass was sent for pathology. She was discharged in good condition three days after the thyroidectomy with a follow-up a week later.

**Figure 3:**
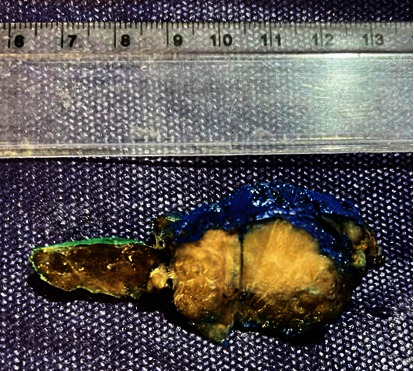
Cut section of the thyroid shows an ill-defined creamy firm mass in the right lobe.

**Figure 4:**
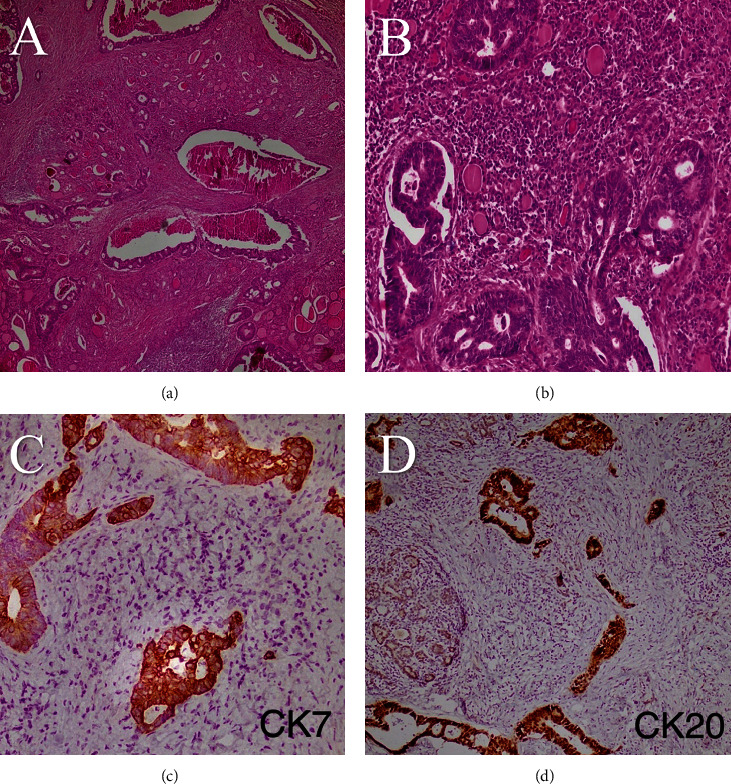
Microscopic sections show metastatic glands of colon adenocarcinoma, infiltrating between thyroid follicles: (a) H&E ×40 and (b) (H&E ×200). The metastatic gland shows immunoreactivity for CK7 (c) and CK20 (d).

**Figure 5:**
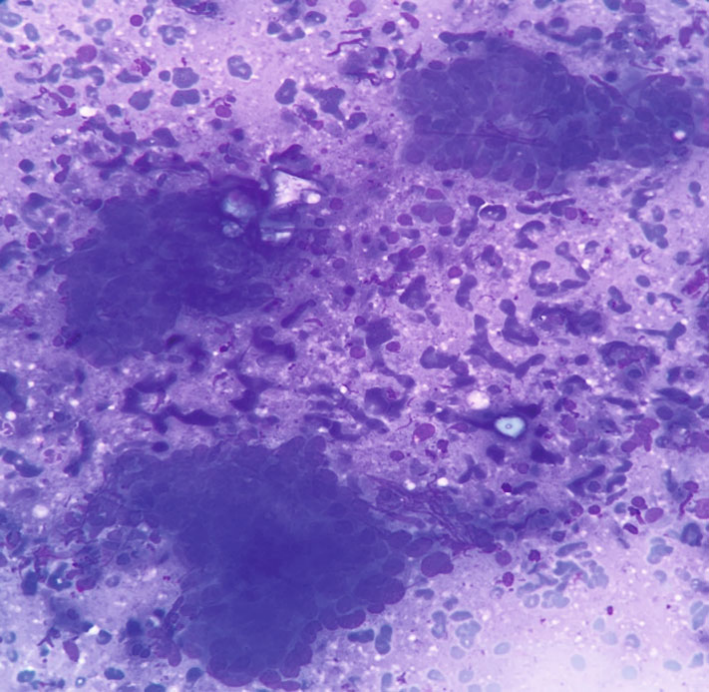
Cytology smear shows moderate number of isolated and clusters of highly atypical cells.

Pathological examination of total thyroidectomy revealed metastatic adenocarcinoma with colon origin. The mass was in the right lobe with the size of 2.7 × 2 × 1.2 cm, and the resected margin was free of tumor. Also, the lymphovascular invasion was seen, and the nonneoplastic thyroid showed Hashimoto's thyroiditis. The immunohistochemistry evaluation confirmed the diagnosis: positive for CK7, CK20, and CDX2 and negative for TTF-1 (Figures 3–5).

## 3. Discussion

Thyroid dysfunction rarely occurs among patients with thyroid metastasis and is asymptomatic. It is not a common clinical presentation for a nonthyroidal malignancy to metastasize to the thyroid gland. This is observed only in 1.4-3% of autopsied cases; also, they are mostly metastases from lung cancer [5]. There are two acceptable hypotheses discussing the rarity of thyroid disease. It may either be because of abundant arterial blood supply to the thyroid and rapid blood flow, which disturbs the possibility of tumor cell adhesion and implantation or high oxygen saturation and high amount of iodine in the thyroid preventing the process of tumor cell establishment and development in the thyroid gland [6].

Imaging studies of the neck are not included routinely in follow-up examinations of colorectal cancer, but international colon cancer guidelines suggested that in case of proven metastatic synchronous adenocarcinoma, such as in our case who had metastatic adenocarcinoma to the lung, a PET scan should be included in the follow-up [7]. In a study by Froylich et al., it was shown that thyroid metastases were diagnosed between six months and eight years after colectomy [8]. The same timeline applies to the case presented with thyroid metastasis presenting eight years after hemicolectomy.

The rise in tumor markers can be helpful to confirm whether it is primary thyroid cancer or metastatic. TTF-1 and Tg are thyroid-specific markers, while CDX2 is a tumor marker of gastrointestinal adenocarcinomas, especially colorectal cancer. In our case, TTF-1 was negative while CDX 2 was positive with a positive past medical history of colorectal cancer; thus, the tumor markers are in favor of metastatic adenocarcinoma from colon cancer [9–11].

Although thyroid nodules can be detected in imaging studies, such as PET-CT, FNA is needed to determine whether the lesion is a primary malignancy or metastatic. However, in our case, FNA showed papillary thyroid carcinoma, which was a misdiagnosis, and the final diagnosis showing the metastatic region of the mass was made postoperatively in our patient [12].

After the nature of the thyroid mass is revealed, surgical treatment and chemotherapy are the management of thyroid metastasis; the extent of metastatic involvement and the patient's condition must be concerned before starting these treatments. Since there was a lymphovascular invasion in our case, lymph node dissection was done [12, 13]. Also, in the presence of bilateral metastatic thyroid cancer or large tumor size, total thyroidectomy is recommended [14]. Thyroidectomy was done for our patient due to the large size of the mass (2.7 × 2 × 1.2 cm).

## 4. Conclusion

Thyroid metastasis from colorectal cancer rarely occurs, but in case of happening, it would not get diagnosed until the latent course of the disease because they have an asymptomatic behavior, and thyroid studies are not performed routinely. Therefore, clinicians must be attentive to patients' thyroid in the presence of a history of previous colon cancer. A solitary thyroid nodule with a past medical history of cancer should raise the suspicion of thyroid metastasis. Concerning treatment, surgical treatment is the priority due to its effect on patients' survival and improvement of their life quality.

## Data Availability

All data regarding this case report has been reported in the manuscript. Please contact the corresponding author in case of requiring any further information.

## Abbreviations

CT: Computed tomography
FNA: Fine needle aspiration
PET: Positron emission tomography (PET)
PTE: Pulmonary thromboembolism.
